# Associations of Maternal Complaints to Levator Ani Muscle Trauma within 9 Months after Vaginal Birth: A Prospective Observational Cohort Study

**DOI:** 10.1155/2022/4197179

**Published:** 2022-09-05

**Authors:** N. Kimmich, J. Birri, A. Richter, R. Zimmermann, M. Kreft

**Affiliations:** Division of Obstetrics, University Hospital of Zurich, Raemistrasse 100, 8091 Zurich, Switzerland

## Abstract

**Introduction:**

Pelvic floor trauma in the form of partial or complete avulsions of the levator ani muscle (LAM) affects 6-42% of women after vaginal birth and can cause tremendous long-term morbidity. Many studies assessed morphological pelvic floor trauma after childbirth but lacked to evaluate women's associated short-term complaints. A proper assessment of trauma and subjective complaints after birth could help to assess possible associations between them and their relevance to women's daily life. Therefore, we aimed to assess women's complaints within the first months after birth in association to their LAM trauma.

**Materials and Methods:**

Between 3/2017 and 4/2019, we prospectively evaluated vaginal births of 212 primiparous women with singletons in vertex presentation ≥ 36 + 0 gestational weeks for levator ani muscle (LAM) trauma by translabial ultrasound, for pelvic organ prolapse by clinical examination, and for urogynecological complaints using questionnaires 1-4 days (P1), 6-10 weeks (P2), and 6-9 months (P3) after birth. The questionnaires were self-designed but oriented to and modified from validated questionnaires. Women's complaints were evaluated for P1-P3 according to their LAM trauma state.

**Results:**

At P1, 67% of women showed an intact LAM, whereas 14.6% presented a hematoma, 6.6% a partial avulsion (PAV), and 11.8% a complete avulsion (CAV). At P2, 75.9% showed an intact LAM, 9.9% a PAV, and 14.2% a CAV. At P3, 72.9% of women with a LAM trauma in P1 and/or P2 were assessed with 21.6% being intact and 39.2% having a PAV and CAV, respectively. Obstetrical and baseline characteristics differed slightly between the groups. When comparing the time before and during pregnancy with the time after childbirth, birth itself affected women's complaints in all LAM state groups, but the presence of a LAM trauma, especially a CAV, had more negative effects.

**Conclusions:**

Vaginal birth changes the anatomical structure of the maternal birth canal and genital tract, and it alters women's perceptions and body function. In our study, LAM trauma did not change these effects tremendously within the first months. Therefore, other maternal, fetal, and obstetrical factors need consideration for the explanation of maternal complaints, in addition to long-term effects of trauma and dysfunction of the LAM and other birth canal structures.

## 1. Introduction

Pelvic floor trauma in the form of partial or complete avulsions of the levator ani muscle (LAM) affects 6-42% of women after vaginal birth [1–7]. LAM defects, especially complete avulsions of the LAM, are associated with genital prolapse in later life and can cause tremendous short- and long-term morbidity in affected women, including pain, sexual dysfunction, incontinence, prolapse, and psychological distress [8–14]. In the past years, many studies were published about the assessment of morphological pelvic floor trauma after childbirth. Hereby, LAM avulsions were diagnosed most reliably by 4-dimensional (4D) translabial ultrasound (TLUS) as the standard diagnostic tool [11, 15–18]. However, little emphasis has been laid on women's complaints associated with these changes, especially within the first months after birth. A proper assessment and documentation of trauma of women's complaints after birth could help to assess possible associations between morphological changes and subjective complaints and their relevance to women's daily life in order to identify women of risk for impairment later on [19]. Therefore, the aim of our prospective study was to assess different LAM trauma states of primiparous women by 4D ultrasound examination and to evaluate their subjective complaints by questionnaires at different times within 9 months after birth according to their allocation to different LAM trauma states.

## 2. Materials and Methods

We performed a prospective observational cohort study at our tertiary care center in Switzerland between 3/2017 and 4/2019. The study was approved by the Local Ethical Board of the District under the registration BASEC-No. 2016-00908. Additionally, all study participants gave their written informed consent for the study.

Nulliparous women ≥ 18 years of age with singleton pregnancies in vertex presentation ≥ 36 + 0 gestational weeks (gw), who planned a vaginal birth in our institution, were recruited in advance. We excluded multiple pregnancies, deliveries before 36 + 0 gw, planned cesarean sections, fetal transverse or breech positions, fetal malformations, maternal connective tissue diseases, maternal history of vaginal, perineal or vulvar surgery, maternal history of preexisting daily urine or anal incontinence, and difficulties in communication due to insufficient communication in German or English. Clinical data were obtained from the institutional obstetric database (Perinat version 6.1.9.45).

First, women underwent a urogynecological examination by a well-trained senior physician of the department for clinical pelvic organ prolapse at P2 and P3, quantified by the POP-Q classification system of the International Urogynecological Association (POP-Q stages 0-4). No such an assessment was performed directly after birth at P1 due to women's discomfort by any kind of birth tears, hematoma, or swelling. Women with POP-Q stages 0 and 1 of the anterior genital compartment were assigned to the “no anterior prolapse” group, women with POP-Q stages 0 and 1 of the posterior compartment to the “no posterior prolapse” group, and women with POP-Q stages 0 and 1 of the central compartment to the “no central prolapse” group. Women with POP-Q stages 2-4 of the anterior, posterior, and central compartment were assigned to the “anterior prolapse,” “posterior prolapse,” and “central prolapse” group, respectively, for final evaluation. This allocation was used as a clinical relevant prolapse is generally accepted with POP-Q stages 2 and higher.

Second, all women that finally gave birth vaginally were additionally evaluated for their LAM trauma state 1-4 days (P1) and 6-10 weeks after birth (P2). LAM state was assessed by 4D translabial ultrasound by two well-trained pelvic floor sonographers. Women were asked to empty their bladder before the examination and were placed in lithotomy position. A covered 4D abdominal probe of 4 to 7 MHz (Voluson S10, GE Healthcare, Zipf, Austria) was placed between the labia at the posterior fourchette. Acquisition of 4D tomographic volumes and their interpretation were performed as described by Dietz et al. [15, 17]. Tomographic ultrasound imaging (TUI) was performed with 2.5 mm slice intervals, from 5 mm below to 12.5 mm above the plane of minimal hiatal dimensions. All images were reviewed and optimized in a second step with the software 4D View 7.0 (GE Healthcare, Zipf, Austria). Two reviewers then classified LAM states into intact, hematoma, partial avulsion (PAV), or complete avulsion (CAV) [15, 16]. In case of a discordant diagnosis between the two investigators, volumes were reexamined together and the diagnosis was verified. A partial avulsion was stated; when there was continuity of some part of the muscle to the ramus inferior of the os pubis and a complete avulsion, there was a complete discontinuity of the muscle to the pelvic sidewall. An-/hypoechogenic lesion inside the muscle with intact muscle tissue on both sides around was attributed to a hematoma. All women diagnosed with a LAM trauma (hematoma, PAV, and CAV) at P1 and/or P2 were invited for another evaluation 6-9 months after birth (P3).

To assess subjective impairment and complaints after birth and in comparison to the time before and during pregnancy, every woman was asked to complete a questionnaire with multiple questions about their urogynecological condition at each assessment period directly after the clinical examination (Table 1). The used questionnaire was self-designed but oriented to and modified from validated questionnaires, such as the King's Health Questionnaire, the Pelvic Floor Distress Inventory, the Pelvic Floor Impact Questionnaire, and the “Deutscher Beckenboden-Fragebogen.”

At P1, women were asked to answer questions about their condition at either the time before or during pregnancy, respectively (Table 1, Nos. 1-7), to control for preexisting urogynecological complaints and disorders with 3 possible answers (“never,” “sometimes,” or “daily/always”). Then, they were asked 12 questions (Table 1, Nos. 8-19) about their current urogynecological condition. The same 12 questions were asked at the other two assessment periods P2 and P3. Women taking part in the assessment period P2 and P3 additionally completed another nine questions (Table 1, Nos. 20-28).

Part of the questions at P1-P3 had to be answered with either “yes” or “no,” others with a graduation from 1 to 7 on a scale. The graduation 4 in the middle of the scale represented the situation of “unchanged” after birth compared to the time before pregnancy. The graduation 1-3 to the left represented “less often/tighter/weaker/drier/reduced,” for example “little less” (grade 3), “less” (grade 2), and “clearly/much less” (grade 1). The graduation to the right side with 5 to 7 represented “more often/wider/stronger/wetter/better/increased,” with, for example, “little more” (grade 5), “more” (grade 6), and “clearly/much more” (grade 7). For final analysis of the questionnaires, the answer grades 1 and 2 were summarized as “less often/tighter/weaker/drier/reduced,” the answer categories 3 to 5 as “unchanged,” and the categories 6 and 7 as “more often/wider/stronger/wetter/better/increased.”

The answers of women to the mentioned questions were evaluated for the different periods P1-P3 according to their allocation to the different LAM state groups (“intact LAM” vs. “hematoma” vs. “PAV” vs. “CAV” at P1 and “intact LAM” vs. “PAV” vs. “CAV” at P2 and P3, respectively).

Statistical analysis was performed using the statistical software package SPSS version 25.0 (IBM SPSS, Armonk, New York, USA). Student's *t*-test was used to compare continuous variables, and chi-square test was used for categorical variables.

## 3. Results

**Figure 1:**
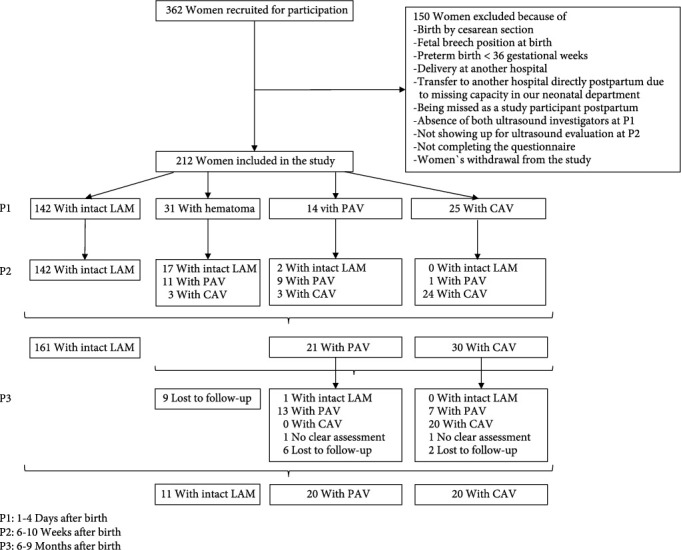
Study flow chart of the study cohort.

362 nulliparous women gave their written informed consent for participation, of whom 150 women had to be excluded from the study later on. The reasons for exclusion were birth by cesarean section, fetal breech position at birth, preterm birth < 36 gestational weeks, delivery at another hospital, and immediate transfer to another hospital due to missing capacity in our neonatal department, because women were missed being a study participant postpartum, because of absence of both ultrasound investigators at P1, because of women's decision to withdraw from the study, and because they did not show up for the ultrasound evaluation after 6-10 weeks or they refused to fill out the questionnaire at P1. Finally, 212 data sets remained for analysis (Figure 1).

### 3.1. Levator Ani Muscle State

The distribution of the LAM states of the study cohort at the P1-P3 can be seen in Figure 1.

At P1, 67% (*n* = 142) of women showed a completely intact LAM. A hematoma inside the LAM was diagnosed in 14.6% (*n* = 31), a PAV in 6.6% (*n* = 14), and a CAV in 11.8% (*n* = 25) of women.

At P2, 75.9% (*n* = 161) of women showed an intact LAM, as an addition to the 142 women with intact LAM at P1 54.8% of women with an initial hematoma and 14.3% of women with an initial PAV had an intact LAM at P2. A PAV was found in 9.9% (*n* = 21) of women at P2, as 64.3% of PAVs at P1 remained as a PAV, and 35.5% of hematomas and 4% of CAVs at P1 turned into a PAV. A CAV in P2 was diagnosed in 14.2% (*n* = 30) of women, as 96.0% remained as a CAV, and 9.7% of hematomas and 21.4% of PAVs in P1 changed into a CAV at P2. None of the hematomas from P1 persisted at P2, as all of them resolved.

Of the 70 women with any kind of LAM trauma in P1 and/or P2, 53 women (75.7%) were available for the examination at P3. Two of those had to be excluded due to poor ultrasound image quality and impossibility to assess the LAM state correctly. Therefore, 51 women showed a clear LAM state at P3, with 21.6% (*n* = 11) being intact and 39.2% (*n* = 20) presenting with a PAV and CAV, respectively.

### 3.2. Baseline Demographic and Obstetric Data

The baseline demographic and obstetric data of the study cohort are shown in Table 2.

Significant differences in the baseline characteristics of the different LAM state groups at P1 were found for the birth mode, epidural anesthesia rates, episiotomy rates, and the duration of the second stage of labor. Women with a PAV or CAV had more often a vacuum-assisted vaginal birth (*p* = 0.022) than the others. Women with a hematoma or CAV had less often an epidural anesthesia (*p* = 0.013). Women with any kind of LAM trauma had twice as often an episiotomy as women with intact LAM (*p* = 0.001) and had a longer duration of the second stage of labor (*p* = 0.018).

At P2, significant differences were found for episiotomy rates, rates of high-grade perineal tears, and labial tears. Women with PAV and CAV had more often an episiotomy (*p* = 0.001), women with CAV had higher rates of high-grade perineal tears (*p* = 0.031), and women with intact LAM showed more often labial tears (0.017).

At P3, significant differences were found for labial tears and anterior prolapse rates, with women with intact LAM showing more often labial tears (*p* = 0.003) and women with CAV suffering more often from an anterior prolapse (*p* = 0.036).

### 3.3. Assessment of Women's Complaints

#### 3.3.1. Assessment regarding the Time before and during Pregnancy

Before pregnancy, 87-100% of women were free from any urinary incontinence, and up to 8.5% complained about urinary incontinence from time to time. During pregnancy, however, 36-46% of women reported urinary incontinence from time to time and up to 7% even daily. The rates for stool incontinence were very low at all times, with a maximum of 4% from time to time during pregnancy. Nevertheless, 29-43% of women suffered from flatus incontinence before pregnancy and even 43-64% during pregnancy. Prolapse symptoms were rare before pregnancy (3-7%) but present in 16-24% of women during pregnancy.

No significant differences between the four LAM state groups were assessed regarding the complaints before and during pregnancy (Supplement 1).

#### 3.3.2. Assessment at 1-4 Days after Birth (P1)

The results for maternal complaints shortly after birth are presented in Table 3.

Significant differences in maternal complaints shortly after birth were found in relation to involuntary stool loss compared to the time before birth (*p* = 0.036) and in relation to the vaginal opening after birth (*p* = 0.037), as can be seen in Table 3.

Women with a PAV were not affected from involuntary stool loss at all. Among the 10 affected women suffering from involuntary stool loss, only the women with a CAV (*n* = 3) were all more often affected compared to the time before birth; all other women showed an unchanged or even improved situation compared to the time before childbirth.

75% of women in the intact LAM group did not feel a change of their vaginal opening after birth in contrast to just about half (51-57%) of the women in the three LAM trauma groups. Only 10% of the women with intact LAM had the feeling of a wider vaginal opening in contrast to up to 28% in the CAV group.

When comparing the first few days after birth with the time during pregnancy, it seems that women complain less about urine incontinence (13-28% vs. 36-51%), little less about flatus incontinence (39-56% vs. 43-64%), and little more about prolapse symptoms (7-48% vs. 16-24%) and stool incontinence (3-12% vs. 3-5%).

### 3.4. Assessment at 6-10 Weeks after Birth (P2)

The results for maternal complaints at P2 after birth are presented in Table 4.

Statistically significant results for the three different LAM state groups (intact vs. PAV vs. CAV) at P2 were found for involuntary gas loss compared to the time before birth (*p* = 0.003) and for pelvic floor strength (*p* = 0.001), as can be seen in Table 4.

The rates of involuntary bowel gas loss did not differ significantly between the LAM state groups at P2 but differed for the groups compared to the time before birth. However, if women complained about involuntary bowel gas loss, 82% of those with an intact LAM had an unchanged situation compared to the time before birth, whereas 46-50% of women in the LAM trauma groups reported a higher frequency of uncontrolled loss of bowel gas. Interestingly, one-third of women in the PAV group even reported an improvement of bowel gas loss.

52-57% of the women in the LAM trauma groups reported about a weaker pelvic floor at P2 compared to the time before birth, in contrast to just 25% of women with a weaker pelvic floor in the intact LAM group. No woman in the LAM trauma groups mentioned a stronger pelvic floor, in contrast to 5% of women in the intact LAM group.

When comparing P2 with P1, it seems that women complain more about urine incontinence (27-33% vs. 13-28%), less about prolapse symptoms (12-27% vs. 7-48%), less about flatus incontinence (24-37% vs. 39-56%), and less about stool incontinence (1.9-5.3% vs. 3-12%).

### 3.5. Assessment at 6-9 Months after Birth (P3)

The results for maternal complaints at P3 after birth are presented in Table 5.

Significant results for the different LAM state groups at P3 were found for the feeling of something squeezing downwards into the vagina (*p* = 0.035), for the frequency of pelvic floor exercise performance (*p* = 0.034), and for satisfaction with sexual intercourse (*p* = 0.040), as can be seen in Table 5.

18-35% of women after vaginal birth had the feeling of something squeezing downwards into the vagina after birth. There were no significant differences regarding this rate between the three LAM state groups. However, in women who described this feeling, all of them described it being apparent more often in the intact LAM group, all of them being unchanged in the PAV group and mainly being unchanged to more often in the CAV group.

Regarding pelvic floor muscle exercise, only half of the women in the PAV group performed some training in contrast to 81-85% in the intact LAM and CAV group, respectively.

85-91% of women had sexual intercourse after birth. 60-83% reported an unchanged situation regarding sexual satisfaction. Nevertheless, 20-29% of women in the intact LAM and PAV group reported a reduced satisfaction compared to just 5.6% in the CAV group, but 20% of women in the intact LAM group had an increased satisfaction in contrast to none in the PAV and CAV group.

When comparing the women in the different LAM state groups at P3 with P2, it seems that women with intact LAM report less often about involuntary urine loss (18 vs. 28%), but women with a LAM trauma complain more often about urine incontinence (40-45% vs. 27-33%). Rates for stool incontinence are comparable between the groups and assessment periods. Bowel gas incontinence was less present at P3 compared to P2 (18-30% vs. 24-37%), as were prolapse symptoms (9-40% vs. 12-23%). Women at P3 performed more often pelvic floor exercise compared to P2 (50-85% vs. 57-63%) and had sexual intercourse in 85-91% of cases in contrast to just 30-39% at P2.

## 4. Discussion

Vaginal birth changes the anatomical structure of the maternal birth canal and genital tract, and it alters women's perceptions and body function. In our study, LAM trauma does not seem to change urogynecological perceptions of women after vaginal birth tremendously within the first months, although there arise some significant differences in women with and without LAM trauma. Therefore, other maternal, fetal, and obstetrical factors have to be considered for the explanation of maternal complaints besides LAM trauma.

### 4.1. Differences in Baseline Characteristics within the Cohort

For our study cohort, we found significant differences in some of the baseline characteristics of the different LAM state groups, depending on the time after birth. These differences are probably due to an altered biomechanics of birth in the LAM trauma groups compared to women with an intact LAM. Women with a PAV or CAV had more often a vacuum-assisted birth than those women with an intact LAM or hematoma. It remains unclear whether the performance of the vacuum maneuver itself might have caused the damage or if the dimensions of the fetus in relation to the maternal birth canal were more often unfavorable resulting in the need for vacuum-assisted birth. Damage due to the vacuum maneuver itself could have been due to a fast performance of the maneuver with too little time for the tissue to stretch and adapt or due to an unfavorable position and rotation of the child within the birth canal. In a recent publication of our group, we could not evaluate a significant maternal, fetal, or obstetrical factor during vacuum maneuver associated to the incidence of LAM avulsions, but we could not control for the just mentioned factors there [20]. Hence, the relation of the fetus and the birth canal and their adaptations to each other during the birth process might be crucial. The higher rate of episiotomies and perineal tears as well as the longer duration of the second stage of labor in women with LAM trauma probably supports this explanation. An unfavorable biomechanical interaction of the fetus and the birth canal might be associated with a prolonged and more complicated birth process and might lead to more interventions. Additionally, it is known that whenever tears in the posterior compartment or episiotomies are present, less tears in the anterior compartment occur, such as labial tears. Therefore, women with intact LAM and with fewer injuries in the posterior compartment might have had higher rates of labial tears in our cohort. The same was seen at P3 for labial tears.

Women with a hematoma or complete avulsion at P1 had less often an epidural anesthesia in this study. From biomechanical simulation studies, one can conclude that a relaxed pelvic floor during birth with no additional coactivation of the pelvic floor muscles reduces the stretch forces on the LAM, as can be achieved by the application of an epidural anesthesia [21]. A study of Youssef et al. supports this statement, as it showed that a LAM coactivation was associated with a longer second stage of labor in nulliparous women [22]. Hence, an epidural might have been protective regarding pelvic floor trauma in our cohort.

Strong associations for CAV and prolapse symptoms in later life are described in the literature many years after childbirth [13, 23, 24]. We could find such an association already some months after birth in our cohort, with higher anterior prolapse rates in women with CAV.

### 4.2. Assessment of Women's Complaints regarding the Time before and during Pregnancy

No significant differences between the four LAM state groups were assessed regarding the time before and during pregnancy, resembling a homogeneous cohort of women in our study. The incontinence rates found in our cohort are in accordance with the rates in the literature [25–27]. The reported rates of incontinence or prolapse symptoms underline that pregnancy itself with its mechanical and hormonal changes alters the voiding and defecation function and it influences the anatomical organ structures.

### 4.3. Assessment of Women's Complaints 1-4 Days after Birth (P1)

Only women with a CAV were all more often affected regarding involuntary stool loss compared to the time before birth. The higher number of affected women in the CAV group cannot be explained by the presence of a high-grade perineal tear, as the rates did not differ significantly between the groups. An explanation could be an altered anal sphincter and levator ani muscle function due to a possible nerve damage or pelvic floor muscle overdistension within the first days after birth rather than a structural muscle damage due to a more complicated birth in that CAV group as mentioned above. Weidner et al. found a neuropathic injury of the levator ani muscle in 24% to 29% of women at 6 weeks and 6 months after childbirth, respectively [28].

The feeling of a wider vaginal opening was more present in the CVA group compared to the intact group (28% vs. 10%). A wider vaginal opening is caused either by a structural widening, for example, by an avulsion of the LAM or by a functional widening by overdistension of the genital hiatus and the vagina or by neuropathic injury [28]. Besides the structural damage of the LAM in the CAV group, the functional widening by a longer second stage of labor in the LAM trauma groups could explain the higher number of women with the feeling of a wider vagina in the trauma groups. In addition, tissue characteristics and distension capabilities of the individual woman might substantially contribute to the altered body anatomy and perception.

### 4.4. Assessment of Women's Complaints 6-10 Weeks after Birth (P2)

The rates of involuntary bowel gas loss did not differ significantly between the LAM state groups compared to the time before birth, although the rate of high-grade perineal tears was significantly higher in the CAV group. However, if women complained about involuntary bowel gas loss, only 18% of women in the intact LAM group reported a higher frequency of uncontrolled bowel gas loss in contrast to 46-50% of women in the LAM trauma groups. Again, besides the structural damage of the anal sphincter, a functional impairment by for example nerve impairment might be crucial, and functional damage is more likely in complicated births. Interestingly, one-third of women in the PAV group even reported an improvement of bowel gas loss. We do not have any explanation for this phenomenon.

The found weakening of the pelvic floor function of women in the LAM trauma groups can also be well comprehensible, as a defect of the pelvic floor muscle in form of a PAV or CAV negatively affects the performance of the LAM. This association has already been described in the literature as well [24].

### 4.5. Assessment of Women's Complaints 6-9 Months after Birth (P3)

18-35% of women after vaginal birth had the feeling of something squeezing downwards into the vagina after birth. There were no significant differences regarding this rate between the three LAM state groups. However, in women who described this feeling, all of them described it being apparent more often in the intact LAM group, whereas all women in the PAV group and most women in the CAV group described the situation as unchanged. This is interesting, as we would have expected such a complaint more frequently in the CAV group with the higher rate of anterior prolapse, especially as this phenomenon is reported in the literature [23, 29]. Hence, a functional problem of the pelvic floor with “just” overdistension of the LAM instead of avulsion could explain this phenomenon in the intact LAM group. Unfortunately, overdistension of the genital hiatus was not evaluated with this study, so we cannot prove this idea. Besides, the number of women in the groups regarding this topic was very small, and conclusions need to be drawn with great caution.

Although women in the CAV group trained their pelvic floor more frequently, they suffered more often from an anterior prolapse than women in the PAV or intact LAM group. Hence, pelvic floor training cannot completely compensate the anatomical defect set by a CAV but maybe can improve the extent and rate of prolapse in this group.

85-91% of the women in our study returned to sexual intercourse within 6-9 months after birth, which is comparable with other studies showing that 89% of women resume sexual activity within 6 months of giving birth [30]. 20-29% of the women in the intact LAM and PAV group reported about sexual dysfunction and even less in the CAV group with 5.7%. In the literature, some studies report higher rates of sexual function and satisfaction after vaginal birth in comparison to the prepregnancy period, with rates of up to 64% at 6 months after birth [31]. Other studies in contrast report that sexual function after birth is not altered by pelvic floor muscle strength or mode of delivery but by the presence of high-grade perineal tears [30, 32, 33]. The association of sexual function and satisfaction to the presence of a LAM avulsion remains controversial [29, 34].

Complaints about urinary incontinence changed over the course of pregnancy and the postpartum period in our study. 36-51% of women declared urinary incontinence during pregnancy, 13-28% at P1, 27-33% at P2, and 18%-45% at P3, with 18% in the intact LAM group and 40-45% in the LAM trauma groups. The improvement might be due to a natural recovery of the nerve structures of the pelvic floor and bladder, intrinsic improvement of pelvic floor muscle function, and effects of pelvic floor muscle training. In contrast to the higher rate of urinary incontinence in women with LAM trauma in our study, other studies found no differences in stress urinary incontinence after vaginal birth in women with or without LAM trauma [35, 36]. A similar trend and course over time could be seen for flatus incontinence in our study with the same possible explanation as above. Prolapse symptoms in our study were found in 16-24% during pregnancy, in 7-48% at P1, in 12-27% at P2, and in 9-40% at P3, with women in the LAM trauma groups being more often affected, especially women with CAV. However, women with intact LAM were also affected. Therefore, prolapse symptoms are not solely associated to structural LAM defects but also to functional impairments, as, for example, the lower capability to recruit and control pelvic floor muscles.

The strength of our study is definitely the longitudinal, prospective design with antenatal inclusion of the participants into the study, a small dropout rate regarding the LAM assessment 6-10 weeks after birth, and the validated assessment methods. Nevertheless, a limitation is the small number of women with LAM trauma at all assessment periods and the small cohort of women with intact LAM at P3 for comparison. For a more precise evaluation of the possible causes of women's complaints, it would be necessary to check not only for structural LAM trauma but even for functional trauma. The dimensions of the genital hiatus should be examined to detect possible overdistension, and the pelvic floor muscles and the innervating nerves need to be checked for impaired recruitment and function. Besides, the surrounding connective and soft tissue of the pelvis and the birth canal need to be evaluated. Furthermore, it would be worthwhile to find a way to get more insight into the adaptation processes of fetus and birth canal. In the first step, simulation models could help to calculate the maternal bony and soft tissues; the different size relations of the fetus and birth canal; the adaptation processes of these structures to the passing fetus; the adaptations of the fetus to the birth canal including its rotation, head flexion, and head molding; and the different labor durations for the necessary adaptations.

## 5. Conclusions

Vaginal birth changes the anatomical structure of the maternal birth canal and genital tract, and it alters women's body perceptions and function. In our study, LAM trauma did not seem to change the effects of a vaginal birth tremendously within the first months, although there are some significant differences present in women with and without LAM trauma. Besides LAM trauma, other maternal, fetal, and obstetrical factors have to be considered for the explanation of maternal complaints. In addition, either long-term effects of LAM trauma or damage and dysfunction of other tissue structures around the birth canal might contribute to women's impairment.

## Figures and Tables

**Table 1:** Questionnaire for assessing the urogynecological conditions of the study population before and during pregnancy and for the time after birth.

Questions regarding the time before and during pregnancy	Possible answers
(1) I lost urine involuntary/against my will	Never ☐ Sometimes ☐ Daily ☐
(2) I lost stool involuntary/against my will	Never ☐ Sometimes ☐ Daily ☐
(3) I lost gas involuntary/against my will	Never ☐ Sometimes ☐ Daily ☐
(4) I had burning/painful sensations during defecation	Never ☐ Sometimes ☐ Always ☐
(5) I had burning/painful sensations during sexual intercourse	Never ☐ Sometimes ☐ Always ☐
(6) I had a feeling of discomfort/foreign body in the vagina	Never ☐ Sometimes ☐ Daily ☐
(7) I had the feeling of something squeezing downwards into the vagina	Never ☐ Sometimes ☐ Daily ☐

Questions regarding the time after birth	Possible answers
(8) I lost urine involuntary/against my will	Yes ☐ No ☐
(9) Compared to the time before childbirth, I lost urine involuntary/against my will	Less often 1 2 3 4 5 6 7 more often
(10) I lost stool involuntary/against my will	Yes ☐ No ☐
(11) Compared to the time before childbirth, I lost stool involuntary/against my will	Less often 1 2 3 4 5 6 7 more often
(12) I lost gas involuntary/against my will	Yes ☐ No ☐
(13) Compared to the time before childbirth, I lost bowel gas involuntary/against my will	Less often 1 2 3 4 5 6 7 more often
(14) I have a feeling of discomfort/foreign body in the vagina	Yes ☐ No ☐
(15) Compared to the time before childbirth, I have a feeling of discomfort/foreign body in the vagina	Less often 1 2 3 4 5 6 7 more often
(16) I have the feeling of something squeezing downwards into the vagina	Yes ☐ No ☐
(17) Compared to the time before childbirth, I have the feeling of something squeezing downwards into the vagina	Less often 1 2 3 4 5 6 7 more often
(18) Compared to the time before childbirth, I have the feeling that my vaginal opening is	Tighter/closer 1 2 3 4 5 6 7 wider
(19) Compared to the time before childbirth, I have the feeling that my pelvic floor is	Weaker 1 2 3 4 5 6 7 stronger
(20) I do pelvic floor exercise	Yes ☐ No ☐
(21) Compared to the time before delivery, I do pelvic floor exercise	Less frequently 1 2 3 4 5 6 7 more frequently
(22) I already had sexual intercourse after delivery	Yes ☐ No ☐
(23) Compared to the time before delivery, my vagina is	Drier 1 2 3 4 5 6 7 wetter
(24) Sexual intercourse is painful	Yes ☐ No ☐
(25) Compared to the time before delivery, sexual intercourse is painful	Less frequently 1 2 3 4 5 6 7 more frequently
(26) Compared to the time before delivery, my orgasm capability is	Worse 1 2 3 4 5 6 7 better
(27) Compared to the time before delivery, my sensation in the vagina during sexual intercourse is	Reduced 1 2 3 4 5 6 7 increased
(28) Compared to the time before delivery, my satisfaction with sexual intercourse is	Reduced 1 2 3 4 5 6 7 increased

The answer “4” represented an unchanged situation after birth compared to the time before pregnancy.

**Table 2:** Baseline demographic and obstetric data of the study cohort according to their allocation to their LAM trauma state and to the time of evaluation at P1-P3.

	P1 (*n* = 212)					P2 (*n* = 212)				P3 (*n* = 51)			
	Intact *n* = 142 (67%)	Hematoma *n* = 31 (14.6%)	PAV *n* = 14 (6.6%)	CAV *n* = 25 (11.8%)	*p* value	Intact *n* = 161 (75.9%)	PAV *n* = 21 (9.9%)	CAV *n* = 30 (14.2%)	*p* value	Intact *n* = 11 (21.6%)	PAV *n* = 20 (39.2%)	CAV *n* = 20 (39.2%)	*p* value
Ethnicity					0.584				0.781				0.820
(i) Caucasian	120 (84.5)	27 (87.1)	11 (78.6)	22 (88.0)		136 (84.5)	18 (85.7)	26 (86.7)		10 (90.9)	18 (90.0)	17 (85.0)	
(ii) Asian	5 (3.5)	0 (0.0)	0 (0.0)	0 (0.0)		5 (3.1)	0 (0.0)	0 (0.0)		0 (0.0)	0 (0.0)	0 (0.0)	
(iii) Mediterranean	7 (4.9)	2 (6.5)	1 (7.1)	2 (8.0)		8 (5.0)	2 (9.5)	2 (6.7)		0 (0.0)	1 (5.0)	2 (10.0)	
(iv) Afro-Caribbean	6 (4.2)	0 (0.0)	0 (0.0)	0 (0.0)		6 (3.7)	0 (0.0)	0 (0.0)		0 (0.0)	0 (0.0)	0 (0.0)	
(v) Oriental	4 (2.8)	2 (6.5)	2 (14.3)	1 (4.0)		6 (3.7)	1 (4.8)	2 (6.7)		1 (9.1)	1 (5.0)	1 (5.0)	
Mode of delivery					0.022^∗^				0.085				0.617
(i) Spontaneous vaginal	113 (79.6)	24 (77.4)	6 (42.9)	18 (72)		127 (89.4)	12 (57.1)	22 (73.3)		7 (63.6)	11 (55.0)	14 (70.0)	
(ii) Vacuum-assisted vaginal	29 (20.4)	7 (22.6)	8 (57.1)	7 (28)		34 (10.6)	9 (42.9)	8 (26.7)		4 (36.4)	9 (45.0)	6 (30.0)	
Epidural analgesia					0.013^∗^				0.202				0.091
(i) Yes	89 (62.7)	14 (45.2)	11 (78.6)	9 (36.0)		98 (60.9)	12 (57.1)	13 (43.3)		4 (36.4)	14 (70.0)	8 (40.0)	
(ii) No	53 (37.3)	17 (54.8)	3 (21.4)	16 (64.0)		63 (39.1)	9 (42.9)	17 (56.7)		7 (63.6)	6 (30.0)	12 (60.0)	
Episiotomy					0.001^∗^				0.001^∗^				0.493
(i) Yes	37 (26.1)	18 (58.1)	7 (50.0)	12 (48.0)		46 (28.6)	14 (66.7)	14 (46.7)		5 (45.5)	10 (50.0)	12 (60.0)	
(ii) No	105 (73.9)	13 (41.9)	7 (50.0)	13 (52.0)		115 (71.4)	7 (23.3)	16 (53.3)		6 (54.5)	10 (50.0)	8 (40.0)	
Low-grade perineal tear					0.264				0.354				0.222
(i) Yes	56 (39.4)	8 (25.8)	6 (42.9)	6 (24.0)		62 (38.5)	6 (28.6)	8 (26.7)		3 (27.3)	9 (45.0)	4 (20.0)	
(ii) No	86 (60.6)	23 (74.2)	8 (57.1)	19 (76.0)		99 (61.5)	15 (71.4)	22 (73.3)		8 (72.7)	11 (55.0)	16 (80.0)	
High-grade perineal tear					0.142				0.031^∗^				0.454
(i) Yes	1 (0.7)	0 (0.0)	1 (7.1)	1 (4.0)		1 (0.6)	0 (0.0)	2 (6.7)		0 (0.0)	0 (0.0)	1 (5.0)	
(ii) No	141 (99.3)	31 (100.0)	13 (92.9)	24 (96.0)		160 (99.4)	21 (100.0)	28 (93.3)		11 (100.0)	20 (100.0)	19 (95.0)	
Vaginal tear					0.781				0.815				0.632
(i) Yes	72 (50.7)	18 (58.1)	6 (42.9)	12 (48.0)		84 (52.2)	10 (47.6)	14 (46.7)		6 (54.5)	12 (60.0)	8 (40.0)	
(ii) No	70 (49.3)	13 (41.9)	8 (57.1)	13 (52.0)		77 (47.8)	11 (52.4)	16 (53.3)		5 (45.5)	8 (40.0)	12 (60.0)	
Labial tear					0.155				0.017^∗^				0.003^∗^
(i) Yes	48 (33.8)	8 (25.8)	2 (14.3)	4 (16.0)		55 (34.2)	2 (9.5)	5 (16.7)		6 (54.5)	1 (5.0)	3 (15.0)	
(ii) No	94 (66.2)	23 (74.2)	12 (85.7)	21 (84.0)		106 (65.8)	19 (90.5)	25 (83.3)		5 (45.5)	19 (95.0)	17 (85.0)	
Anterior prolapse	Not evaluated	Not evaluated	Not evaluated	Not evaluated					0.051				0.036^∗^
(i) Yes						7 (4.3)	2 (9.5)	5 (16.7)		0 (0.0)	0 (0.0)	4 (20.0)	
(ii) No						140 (87.0)	18 (85.7)	24 (80.0)		10 (90.9)	19 (95.0)	15 (75.0)	
(iii) Missing data						14 (8.7)	1 (4.8)	1 (3.3)		1 (9.1)	1 (5.0)	1 (5.0)	
Posterior prolapse	Not evaluated	Not evaluated	Not evaluated	Not evaluated					0.845				0.471
(i) Yes						1 (0.6)	0 (0.0)	0 (0.0)		0 (0.0)	1 (5.0)	0 (0.0)	
(ii) No						145 (90.1)	20 (95.2)	29 (96.7)		9 (81.8)	18 (90.0)	19 (95.0)	
(iii) Missing data						15 (9.3)	1 (4.8)	1 (3.3)		2 (18.2)	1 (5.0)	1 (5.0)	
Central prolapse	Not evaluated	Not evaluated	Not evaluated	Not evaluated					0.708				0.215
(i) Yes						2 (1.2)	0 (0.0)	0 (0.0)		0 (0.0)	0 (0.0)	2 (10.0)	
(ii) No						144 (89.4)	21 (100.0)	29 (96.7)		9 (81.8)	19 (95.0)	17 (85.0)	
(iii) Missing data						15 (9.3)	0 (0.0)	1 (3.3)		2 (18.2)	1 (5.0)	1 (5.0)	
Body mass index in kg/m^2^	23.0 ± 4.0	22.4 ± 3.8	21.7 ± 3.6	21.6 ± 2.5	0.329	22.9 ± 3.9	20.9 ± 3.0	22.4 ± 3.5	0.163	21.3 ± 3.1	23.3 ± 4.3	21.0 ± 2.2	0.136
Age in years	33.0 ± 3.9	33.3 ± 3.9	34.2 ± 4.9	33.6 ± 3.9	0.661	32.9 ± 3.9	33.5 ± 4.6	34.5 ± 3.7	0.142	32.1 ± 4.5	34.3 ± 4.3	33.8 ± 3.5	0.362
Gestational age in days	279 ± 8.2	278 ± 8.9	279 ± 7.5	279 ± 7.5	0.943	279 ± 8.2	277 ± 8.4	279 ± 7.5	0.434	281 ± 5.2	280 ± 7.1	278 ± 8.1	0.576
Duration of the second stage of labor in min	106 ± 61	134 ± 61	144 ± 59	128 ± 65	0.018^∗^	109 ± 62	133 ± 62	132 ± 63	0.072	127 ± 61	150 ± 66	132 ± 58	0.552
Pushing duration of the second stage of labor in min	54 ± 40	65 ± 40	81 ± 37	64 ± 42	0.071	56 ± 40	67 ± 40	66 ± 40	0.241	67 ± 45	76 ± 44	65 ± 38	0.704
Neonatal weight	3332 ± 397	3338 ± 520	3382 ± 347	3458 ± 342	0.545	3348 ± 412	3311 ± 448	3397 ± 361	0.746	3546 ± 440	3382 ± 416	3448 ± 336	0.543
Neonatal head circumference	34.8 ± 1.4	34.9 ± 1.4	35.1 ± 1.5	35.2 ± 1.1	0.457	34.8 ± 1.3	35.1 ± 1.4	34.9 ± 1.3	0.617	35.0 ± 1.4	35.2 ± 1.5	35.2 ± 1.2	0.939

Values in *n* (%), mean ± SD, PAV = partial avulsion; CAV = complete avulsion. ^∗^Statistical significance.

**Table 3:** Pelvic floor symptoms between the four different levator ani muscle state groups 1-4 days after birth (P1).

Questions	Intact *n* = 142 (67%)	Hematoma *n* = 31 (14.6%)	PAV *n* = 14 (6.6%)	CAV *n* = 25 (11.8%)	*p* value
(1) I lose urine involuntary/against my will					0.510
(i) Yes	37 (26.1)	4 (12.9)	4 (28.6)	7 (28.0)	
(ii) No	98 (69.0)	24 (77.4)	10 (71.4)	17 (68.0)	
(iii) Missing data	7 (4.9)	3 (9.7)	0 (0.0)	1 (4.0)	
(2) Compared to the time before childbirth, I lose urine involuntary/against my will					0.676
(i) Less often	2 (5.4)	1 (25.0)	0 (0.0)	0 (0.0)	
(ii) Unchanged	16 (43.2)	2 (50.0)	1 (25.0)	3 (42.9)	
(iii) More often	12 (32.4)	1 (25.0)	2 (50.0)	4 (57.1)	
(iv) Missing data	7 (4.9)	0 (0.0)	1 (25.0)	1 (12.5)	
(3) I lose stool involuntary/against my will					0.337
(i) Yes	6 (4.2)	1 (3.2)	0 (0.0)	3 (12.0)	
(ii) No	125 (88.0)	27 (87.1)	13 (92.9)	22 (88.0)	
(iii) Missing data	11 (7.7)	3 (9.7)	1 (7.1)	0 (0.0)	
(4) Compared to the time before childbirth, I lose stool involuntary/against my will					0.036^∗^
(i) Less often	1 (16.7)	0 (0.0)	Not applicable	0 (0.0)	
(ii) Unchanged	5 (83.3)	1 (100.0)		0 (0.0)	
(iii) More often	0 (0.0)	0 (0.0)		3 (100.0)	
(iv) Missing data	0 (0.0)	0 (0.0)		0 (0.0)	
(5) I lose gas involuntary/against my will					0.225
(i) Yes	55 (38.7)	16 (51.6)	6 (42.9)	14 (56.0)	
(ii) No	77 (54.2)	11 (35.5)	7 (50.0)	10 (40.0)	
(iii) Missing data	10 (7.0)	4 (12.9)	1 (7.1)	1 (4.0)	
(6) Compared to the time before childbirth, I lose gas involuntary/against my will					0.368
(i) Less often	1 (1.8)	1 (6.3)	0 (0.0)	1 (7.1)	
(ii) Unchanged	39 (70.9)	9 (56.3)	3 (50.0)	5 (35.7)	
(iii) More often	15 (27.3)	6 (37.5)	3 (50.0)	7 (50.0)	
(iv) Missing data	0 (0.0)	0 (0.0)	0 (0.0)	1 (7.1)	
(7) I have a feeling of discomfort/foreign body in the vagina					0.657
(i) Yes	46 (32.4)	11 (35.5)	5 (35.7)	12 (48.0)	
(ii) No	85 (59.9)	16 (51.6)	8 (57.1)	13 (52.0)	
(iii) Missing data	11 (7.7)	4 (12.9)	1 (7.1)	0 (0.0)	
(8) Compared to the time before childbirth, I have a feeling of discomfort/foreign body in the vagina					0.236
(i) Less often	2 (4.3)	0 (0.0)	0 (0.0)	0 (0.0)	
(ii) Unchanged	17 (37.0)	2 (18.2)	3 (60.0)	1 (8.3)	
(iii) More often	23 (50.0)	8 (72.7)	2 (40.0)	10 (83.3)	
(iv) Missing data	4 (8.7)	1 (9.1)	0 (0.0)	1 (8.3)	
(9) I have the feeling of something squeezing downwards into the vagina					0.396
(i) Yes	24 (16.9)	2 (6.5)	2 (14.3)	6 (24.0)	
(ii) No	109 (76.8)	26 (83.9)	12 (71.4)	19 (76.0)	
(iii) Missing data	9 (6.3)	3 (9.7)	0 (0.0)	0 (0.0)	
(10) Compared to the time before childbirth, I have the feeling of something squeezing downwards into the vagina					0.827
(i) Less often	3 (12.5)	0 (0.0)	0 (0.0)	0 (0.0)	
(ii) Unchanged	12 (50.0)	1 (50.0)	1 (50.0)	2 (33.3)	
(iii) More often	8 (33.3)	1 (50.0)	1 (50.0)	4 (66.7)	
(iv) Missing data	1 (4.2)	0 (0.0)	0 (0.0)	0 (0.0)	
(11) Compared to the time before childbirth, I have the feeling that my vaginal opening is					0.037^∗^
(i) tighter/closer	7 (4.9)	3 (9.7)	3 (21.4)	3 (12.0)	
(ii) unchanged	107 (75.4)	16 (51.6)	8 (57.1)	14 (56.0)	
(iii) wider	15 (10.6)	6 (19.4)	2 (14.3)	7 (28.0)	
(iv) missing data	13 (9.2)	6 (19.4)	1 (7.1)	1 (4.0)	
(12) Compared to the time before childbirth, I have the feeling that my pelvic floor is					0.055
(i) weaker	47 (33.1)	14 (45.2)	5 (35.7)	17 (68.0)	
(ii) unchanged	78 (54.9)	12 (38.7)	8 (57.1)	8 (32.0)	
(iii) stronger	6 (4.2)	0 (0.0)	0 (0.0)	0 (0.0)	
(iv) missing data	11 (7.7)	5 (16.1)	1 (7.1)	0 (0.0)	

Values in *n* (%), PAV = partial avulsion; CAV = complete avulsion. ^∗^Statistical significance.

**Table 4:** Pelvic floor symptoms between the three different levator ani muscle state groups 6-10 weeks after birth (P2).

Questions	Intact *n* = 161 (75.9%)	PAV *n* = 21 (9.9%)	CAV *n* = 30 (14.2%)	*p* value
(1) I loose urine involuntary/against my will				0.871
(i) Yes	45 (28.0)	7 (33.3)	8 (26.7)	
(ii) No	116 (72.0)	14 (66.7)	19 (63.3)	
(iii) Missing data	0 (0.0)	0 (0.0)	3 (10.0)	
(2) Compared to the time before childbirth, I lose urine involuntary/against my will				0.379
(i) Less often	3 (6.7)	1 (14.3)	0 (0.0)	
(ii) Unchanged	25 (55.6)	2 (28.6)	3 (37.5)	
(iii) More often	15 (33.3)	4 (57.1)	5 (62.5)	
(iv) Missing data	2 (4.4)	0 (0.0)	0 (0.0)	
(3) I lose stool involuntary/against my will				0.816
(i) Yes	4 (2.5)	1 (4.8)	1 (3.3)	
(ii) No	157 (97.5)	20 (95.2)	27 (90.0)	
(iii) Missing data	0 (0.0)	0 (0.0)	2 (6.7)	
(4) Compared to the time before childbirth, I lose stool involuntary/against my will				0.558
(i) Less often	2 (50.0)	0 (0.0)	0 (0.0)	
(ii) Unchanged	1 (25.0)	1 (100.0)	1 (100.0)	
(iii) More often	1 (25.0)	0 (0.0)	0 (0.0)	
(iv) Missing data	0 (0.0)	0 (0.0)	0 (0.0)	
(5) I lose gas involuntary/against my will				0.254
(i) Yes	39 (24.2)	6 (28.6)	11 (36.7)	
(ii) No	121 (75.2)	15 (71.4)	17 (56.7)	
(iii) Missing data	1 (0.6)	0 (0.0)	2 (6.7)	
(6) Compared to the time before childbirth, I lose gas involuntary/against my will				0.003^∗^
(i) Less often	3 (7.7)	2 (33.3)	0 (0)	
(ii) Unchanged	32 (82.1)	1 (16.7)	6 (54.5)	
(iii) More often	4 (10.3)	3 (50)	5 (45.5)	
(iv) Missing data	0 (0.0)	0 (0.0)	0 (0.0)	
(7) I have a feeling of discomfort/foreign body in the vagina				0.061
(i) Yes	19 (11.8)	4 (19.0)	8 (26.7)	
(ii) No	141 (87.6)	17 (81.0)	20 (66.7)	
(iii) Missing data	1 (0.6)	0 (0.0)	2 (6.7)	
(8) Compared to the time before childbirth, I have a feeling of discomfort/foreign body in the vagina				0.447
(i) Less often	2 (10.5)	0 (0.0)	0 (0.0)	
(ii) Unchanged	11 (57.9)	2 (50.0)	3 (37.5)	
(iii) More often	5 (26.3)	2 (50.0)	5 (62.5)	
(iv) Missing data	1 (5.2)	0 (0.0)	0 (0.0)	
(9) I have the feeling of something squeezing downwards into the vagina				0.469
(i) Yes	25 (15.5)	3 (14.3)	7 (23.3)	
(ii) No	134 (83.2)	17 (81.0)	21 (70.0)	
(iii) Missing data	2 (1.2)	1 (4.8)	2 (6.7)	
(10) Compared to the time before childbirth, I have the feeling of something squeezing downwards into the vagina				0.088
(i) Less often	4 (16.0)	1 (33.3)	0 (0.0)	
(ii) Unchanged	13 (52.0)	1 (33.3)	1 (14.3)	
(iii) More often	7 (28.0)	1 (33.3)	6 (85.7)	
(iv) Missing data	1 (4.0)	0 (0.0)	0 (0.0)	
(11) Compared to the time before childbirth, I have the feeling that my vaginal opening is				0.059
(i) tighter/closer	9 (5.6)	1 (4.8)	1 (3.3)	
(ii) unchanged	118 (73.3)	17 (81.0)	15 (50.0)	
(iii) wider	29 (18.0)	3 (14.3)	12 (40.0)	
(iv) missing data	5 (3.1)	0 (0.0)	2 (6.7)	
(12) Compared to the time before childbirth, I have the feeling that my pelvic floor is				0.001^∗^
(i) weaker	40 (24.8)	11 (52.4)	17 (56.7)	
(ii) unchanged	106 (65.8)	9 (42.9)	11 (36.7)	
(iii) stronger	8 (5.0)	0 (0)	0 (0)	
(iv) missing data	7 (4.3)	1 (4.8)	2 (6.7)	
(13) I do pelvic floor exercise				0.717
(i) Yes	103 (64.0)	12 (57.1)	19 (63.3)	
(ii) No	55 (34.2)	9 (42.9)	9 (30.0)	
(iii) Missing data	3 (1.9)	0 (0.0)	2 (6.7)	
(14) Compared to the time before delivery, I do pelvic floor exercise				0.062
(i) Less often	8 (7.8)	4 (33.3)	1 (5.3)	
(ii) Unchanged	50 (48.5)	5 (41.7)	8 (42.1)	
(iii) More often	39 (37.9)	3 (25.0)	10 (52.6)	
(iv) Missing data	6 (5.8)	0 (0.0)	0 (0.0)	
(15) I already had sexual intercourse after delivery				0.844
(i) Yes	62 (38.5)	8 (38.1)	9 (30.0)	
(ii) No	97 (60.2)	12 (57.1)	18 (60.0)	
(iii) Missing data	2 (1.2)	1 (4.8)	3 (10.0)	
(16) Compared to the time before delivery, during sexual intercourse, my vagina is				0.290
(i) drier	19 (30.6)	5 (62.5)	5 (55.6)	
(ii) unchanged	38 (61.3)	3 (37.5)	4 (44.4)	
(iii) wetter	4 (6.5)	0 (0.0)	0 (0.0)	
(iv) missing data	1 (1.6)	0 (0.0)	0 (0.0)	
(17) Sexual intercourse is painful				0.285
(i) Yes	38 (61.3)	3 (37.5)	4 (44.4)	
(ii) No	23 (37.1)	5 (62.5)	5 (55.6)	
(iii) Missing data	1 (1.6)	0 (0.0)	0 (0.0)	
(18) Compared to the time before delivery, sexual intercourse is painful				0.537
(i) Less often	0 (0.0)	0 (0.0)	0 (0.0)	
(ii) Unchanged	20 (52.6)	1 (33.3)	3 (75.0)	
(iii) More often	18 (47.4)	2 (66.7)	1 (25.0)	
(iv) Missing data	0 (0.0)	0 (0.0)	0 (0.0)	
(19) Compared to the time before delivery, my orgasm capability is				0.189
(i) worse	5 (8.1)	2 (25.0)	3 (33.3)	
(ii) unchanged	50 (80.6)	6 (75.0)	6 (66.7)	
(iii) better	4 (6.5)	0 (0.0)	0 (0.0)	
(iv) missing data	3 (4.8)	0 (0.0)	0 (0.0)	
(20) Compared to the time before delivery, my sensation in the vagina during sexual intercourse is				0.243
(i) reduced	3 (4.8)	0 (0.0)	2 (22.2)	
(ii) unchanged	52 (83.9)	8 (100.0)	7 (77.8)	
(iii) increased	4 (6.5)	0 (0.0)	0 (0.0)	
(iv) missing data	3 (4.8)	0 (0.0)	0 (0.0)	
(21) Compared to the time before delivery, my satisfaction with sexual intercourse is				0.524
(i) reduced	8 (12.9)	2 (25.0)	3 (33.3)	
(ii) unchanged	48 (77.4)	6 (75.0)	6 (66.7)	
(iii) increased	3 (4.8)	0 (0.0)	0 (0.0)	
(iv) missing data	3 (4.8)	0 (0.0)	0 (0.0)	

Values in *n* (%), PAV = partial avulsion; CAV = complete avulsion. ^∗^Statistical significance.

**Table 5:** Pelvic floor symptoms between the three different levator ani muscle state groups 6-9 months after birth (P3).

Questions	Intact *n* = 11 (21.6%)	PAV *n* = 20 (39.2%)	CAV *n* = 20 (39.2%)	*p* value
(1) I loose urine involuntary/against my will				0.318
(i) Yes	2 (18.2)	9 (45.0)	8 (40.0)	
(ii) No	9 (81.8)	11 (55.0)	12 (60.0)	
(iii) Missing data	0 (0.0)	0 (0.0)	0 (0.0)	
(2) Compared to the time before childbirth, I lose urine involuntary/against my will				0.445
(i) Less often	0 (0.0)	0 (0.0)	0 (0.0)	
(ii) Unchanged	1 (50.0)	8 (88.9)	6 (75.0)	
(iii) More often	(50.0)	1 (11.1)	2 (25.0)	
(iv) Missing data	0 (0.0)	0 (0.0)	0 (0.0)	
(3) I lose stool involuntary/against my will				0.435
(i) Yes	0 (0.0)	1 (5.0)	0 (0.0)	
(ii) No	11 (100.0)	18 (90.0)	20 (100.0)	
(iii) Missing data	0 (0.0)	1 (5.0)	0 (0.0)	
(4) Compared to the time before childbirth, I lose stool involuntary/against my will				
(i) Less often	Not applicable	0 (0.0)	Not applicable	Not applicable
(ii) Unchanged		1 (100.0)		
(iii) More often		0 (0.0)		
(iv) Missing data		0 (0.0)		
(5) I lose gas involuntary/against my will				0.678
(i) Yes	2 (18.2)	4 (20.0)	6 (30.0)	
(ii) No	9 (81.8)	16 (80.0)	14 (70.0)	
(iii) Missing data	0 (0.0)	0 (0.0)	0 (0.0)	
(6) Compared to the time before childbirth, I lose bowel gas involuntary/against my will				0.787
(i) Less often	0 (0.0)	1 (25.0)	1 (16.7)	
(ii) Unchanged	2 (100.0)	3 (75.0)	4 (66.7)	
(iii) More often	0 (0.0)	0 (0.0)	1 (16.7)	
(iv) Missing data	0 (0.0)	0 (0.0)	0 (0.0)	
(7) I have a feeling of discomfort/foreign body in the vagina				0.498
(i) Yes	1 (9.1)	3 (15.0)	5 (25.0)	
(ii) No	10 (90.1)	17 (85.0)	15 (75.0)	
(iii) Missing data	0 (0.0)	0 (0.0)	0 (0.0)	
(8) Compared to the time before childbirth, I have a feeling of discomfort/foreign body in the vagina				0.165
(i) Less often	0 (0.0)	0 (0.0)	0 (0.0)	
(ii) Unchanged	0 (0.0)	3 (100.0)	3 (60.0)	
(iii) More often	1 (100.0)	0 (0.0)	2 (40.0)	
(iv) Missing data	0 (0.0)	0 (0.0)	0 (0.0)	
(9) I have the feeling of something squeezing downwards into the vagina				0.150
(i) Yes	2 (18.2)	2 (10.0)	7 (35.0)	
(ii) No	9 (81.8)	18 (90.0)	13 (65.0)	
(iii) Missing data	0 (0.0)	0 (0.0)	0 (0.0)	
(10) Compared to the time before childbirth, I have the feeling of something squeezing downwards into the vagina				0.035^∗^
(i) Less often	0 (0.0)	0 (0.0)	0 (0.0)	
(ii) Unchanged	0 (0.0)	2 (100.0)	6 (85.7)	
(iii) More often	2 (100.0)	0 (0.0)	1 (14.3)	
(iv) Missing data	0 (0.0)	0 (0.0)	0 (0.0)	
(11) Compared to the time before childbirth, I have the feeling that my vaginal opening is				0.168
(i) tighter/closer	1 (9.1)	1 (5.0)	0 (0.0)	
(ii) unchanged	8 (72.7)	17 (85.0)	12 (60.0)	
(iii) wider	2 (18.2)	2 (10.0)	8 (40.0)	
(iv) missing data	0 (0.0)	0 (0.0)	0 (0.0)	
(12) Compared to the time before childbirth, I have the feeling that my pelvic floor is				0.107
(i) weaker	1 (9.1)	5 (25.0)	10 (50.0)	
(ii) unchanged	10 (90.9)	14 (70.0)	10 (50.0)	
(iii) stronger	0 (0.0)	1 (5.0)	0 (0.0)	
(iv) missing data	0 (0.0)	0 (0.0)	0 (0.0)	
(13) I do pelvic floor exercise				0.034^∗^
(i) Yes	9 (81.2)	10 (50)	17 (85)	
(ii) No	2 (18.8)	10 (50)	3 (15)	
(iii) Missing data	0 (0.0)	0 (0.0)	0 (0.0)	
(14) Compared to the time before delivery, I do pelvic floor exercise				0.399
(i) Less often	2 (22.2)	0 (0.0)	1 (5.9)	
(ii) Unchanged	3 (33.3)	3 (30.0)	6 (35.3)	
(iii) More often	3 (33.3)	6 (60.0)	10 (58.8)	
(iv) Missing data	1 (11.11)	1 (10.0)	0 (0.0)	
(15) I already had sexual intercourse after delivery				0.845
(i) Yes	10 (90.9)	17 (85.0)	18 (90.0)	
(ii) No	1 (9.1)	3 (15.0)	2 (10.0)	
(iii) Missing data	0 (0.0)	0 (0.0)	0 (0.0)	
(16) Compared to the time before delivery, my vagina is				0.543
(i) drier	4 (40.0)	2 (11.8)	5 (27.8)	
(ii) unchanged	6 (60.0)	13 (76.5)	11 (61.1)	
(iii) wetter	0 (0.0)	1 (5.9)1	1 (5.6)	
(iv) missing data	(0.0)	(5.9)	1 (5.6)	
(17) Sexual intercourse is painful				0.676
(i) Yes	3 (30.0)	4 (23.5)	6 (33.3)	
(ii) No	6 (60.0)	13 (76.5)	10 (55.6)	
(iii) Missing data	1 (10.0)	0 (0.0)	2 (11.1)	
(18) Compared to the time before delivery, sexual intercourse is painful				0.296
(i) Less often	0 (0.0)	0 (0.0)	0 (0.0)	
(ii) Unchanged	1 (33.3)	2 (50.0)	5 (83.3)	
(iii) More often	2 (66.7)	2 (50.0)	1 (16.7)	
(iv) Missing data	0 (0.0)	0 (0.0)	0 (0.0)	
(19) Compared to the time before delivery, my orgasm capability is				0.726
(i) worse	1 (10.0)	3 (17.6)	2 (11.1)	
(ii) unchanged	9 (90.0)	14 (82.4)	13 (72.2)	
(iii) better	0 (0.0)	0 (0.0)	1 (5.6)	
(iv) missing data	0 (0.0)	0 (0.0)	2 (11.1)	
(20) Compared to the time before delivery, my sensation in the vagina during sexual intercourse is				0.299
(i) reduced	0 (0.0)	2 (11.8)	1 (5.6)	
(ii) unchanged	10 (100.0)	15 (88.2)	13 (72.2)	
(iii) increased	0 (0.0)	0 (0.0)	2 (11.1)	
(iv) missing data	0 (0.0)	0 (0.0)	2 (11.1)	
(21) Compared to the time before delivery, my satisfaction with sexual intercourse is				0.040^∗^
(i) reduced	2 (20.0)	5 (29.4)	1 (5.6)	
(ii) unchanged	6 (60.0)	12 (70.6)	15 (83.3)	
(iii) increased	2 (20.0)	0 (0.0)	0 (0.0)	
(iv) missing data	0 (0.0)	0 (0.0)	2 (11.1)	

Values in *n* (%), PAV = partial avulsion; CAV = complete avulsion. ^∗^Statistical significance.

## Data Availability

The data sets generated and/or analyzed during the current study are not publicly available due to privacy reasons of the study cohort members and due to the huge amount of data but are available from the corresponding author on reasonable request.

## Supplementary Materials

Supplementary Materials Supplement 1: Pelvic floor symptoms between the four different levator ani muscle state groups for the time before and during pregnancy.
