# Supplementary material for: Associations of Maternal Complaints to Levator Ani Muscle Trauma within 9 Months after Vaginal Birth: A Prospective Observational Cohort Study
Source: J Pregnancy. 2022 Sep 5;2022:4197179. doi: 10.1155/2022/4197179 (PMC9467807; doi:10.1155/2022/4197179)
Supplement: Supplementary Materials — Supplement 1: Pelvic floor symptoms between the four different levator ani muscle state groups for the time before and during pregnancy. [file 4197179.f1.docx]

Supplement 1: Pelvic floor symptoms between the four different levator ani muscle (LAM) state groups for the time before and during pregnancy

| **Questions** | **Before pregnancy** | | | | | **During pregnancy** | | | | |
| --- | --- | --- | --- | --- | --- | --- | --- | --- | --- | --- |
|  | Intact n=142 (67%) | Hematoma n=31 (14.6%) | PAV  n=14 (6.6%) | CAV  n=25 (11.8%) | p-value | Intact n=142 (67%) | Hematoma n=31 (14.6%) | PAV n=14 (6.6%) | CAV n=25 (11.8%) | p-value |
| 1. I lost urine involuntary/ against my will  - Never - Sometimes - Daily - Missing data | 124 (87.3)  12 (8.5)  0 (0.0)  6 (4.2) | 27 (87.1)  2 (6.5)  0 (0.0)  2 (6.5) | 14 (100.0)  0 (0.0)  0 (0.0)  0 (0.0) | 23 (92.0)  2 (8.0)  0 (0.0)  0 (0.0) | .703 | 63 (44.4)  65 (45.8)  8 (5.6)  6 (4.2) | 14 (45.2)  12 (38.7)  2 (6.5)  3 (9.7) | 7 (50.0)  6 (42.9)  1 (7.1)  0 (0.0) | 15 (60.0)  10 (40.0)  0 (0.0)  0 (0.0) | .828 |
| 1. I lost stool involuntary/ against my will  - Never - Sometimes - Daily - Missing data | 133 (93.7)  3 (2.1)  0 (0.0)  6 (4.2) | 29 (93.5)  0 (0.0)  0 (0.0)  2 (6.5) | 13 (92.9)  0 (0.0)  0 (0.0)  1 (7.1) | 25 (100.0)  0 (0.0)  0 (0.0)  0 (0.0) | .682 | 129 (90.8)  6 (4.2)  1 (0.7)  6 (4.2) | 27 (87.1)  1 (3.2)  0 (0.0)  3 (9.7) | 13 (92.9)  0 (0.0)  0 (0.0)  1 (7.1) | 24 (96.0)  1 (4.0)  0 (0.0)  0 (0.0) | .981 |
| 1. I lost gas involuntary/ against my will  - Never - Sometimes - Daily - Missing data | 88 (62.0)  45 (31.7)  3 (2.1)  6 (4.2) | 20 (64.5)  9 (29.0)  0 (0.0)  2 (6.5) | 8 (57.1)  6 (42.9)  0 (0.0)  0 (0.0) | 17 (68.0)  8 (32.0)  0 (0.0)  0 (0.0) | .902 | 54 (38.0)  68 (47.9)  13 (9.1)  7 (4.9) | 11 (35.5)  16 (51.6)  1 (3.2)  3 (9.7) | 8 (57.1)  6 (42.9)  0 (0.0)  0 (0.0) | 9 (36.0)  16 (64.0)  0 (0.0)  0 (0.0) | .341 |
| 1. I had burning/painful sensations during defecation  - never - sometimes - always - Missing data | 106 (74.6)  30 (21.1)  0 (0.0)  6 (4.2) | 20 (64.5)  9 (29.0)  0 (0.0)  2 (6.5) | 11 78.6)  3 (21.4)  0 (0.0)  1 (7.1) | 19 (72.0)  6 (24.0)  0 (0.0)  0 (0.0) | .775 | 93 (65.5)  43 (30.3  0 (0.0)  6 (4.2) | 15 (48.4)  12 (38.7)  1 (3.2)  3 (9.7) | 9 (64.3)  5 (35.7)  0 (0.0)  0 (0.0) | 17 (68.0)  8 (32.0)  0 (0.0)  0 (0.0) | .241 |
| 1. I had burning/painful sensations during sexual intercourse  - never - sometimes - always - Missing data | 90 (63.4)  45 (31.7)  0 (0.0)  7 (4.9) | 17 (54.8)  12 (38.7)  0 (0.0)  2 (6.5) | 12 (85.7)  2 (14.3)  0 (0.0)  0 (0.0) | 15 (56.0)  9 (36.0)  1 (4.0)  0 (0.0) | .107 | 85 (59.9)  48 (33.8)  3 (2.1)  6 (4.2) | 20 (64.5)  6 (19.4)  0 (0.0)  5 (16.1) | 13 (92.9)  1 (7.1)  0 (0.0)  0 (0.0) | 16 (64.0)  8 (32.0)  0 (0.0)  1 (4.0) | .279 |
| 1. I had a feeling of discomfort/ foreign body in the vagina  - never - sometimes - daily - Missing data | 129 (90.8)  6 (4.2)  0 (0.0)  7 (4.9) | 28 (90.3)  1 (3.2)  0 (0.0)  2 (6.5) | 14 (100)  0 (0.0)  0 (0.0)  0 (0.0) | 25 (100.0)  0 (0.0)  0 (0.0)  0 (0.0) | .616 | 119 (83.8)  15 (10.6)  1 (0.7)  7 (4.9) | 25 (80.6)  2 (6.5)  0 (0.0)  4 (12.9) | 13 (92.9)  1 (7.1)  0 (0.0)  0 (0.0) | 22 (88.0)  3 (12.0)  0 (0.0)  0 (0.0) | .983 |
| 1. I had the feeling of something squeezing downwards into the vagina  - never - sometimes - daily - Missing data | 131 (92.3)  4 (2.8)  0 (0.0)  7 (4.9) | 29 (93.5)  0 (0.0)  0 (0.0)  2 (6.5) | 13 (92.9)  1 (7.1)  0 (0.0)  0 (0.0) | 25 (100.0)  0 (0.0)  0 (0.0)  0 (0.0) | .427 | 92 (64.8)  22 (15.5)  3 (2.1)  25 (17.6) | 14 (45.2)  7 (22.6)  0 (0.0)  10 (32.3) | 8 (57.1)  3 (21.4)  1 (7.1)  2 (14.3) | 13 (52.0)  6 (24.0)  0 (0.0)  6 (24.0) | .431 |

Values in n (%), PAV= partial avulsion, CAV= complete avulsion
